# Concordance of Genomic Alterations in Ovarian Cancer Tissues and Circulating-Tumor DNA: A Pilot Study

**DOI:** 10.3390/ijms27031305

**Published:** 2026-01-28

**Authors:** Bowon Kang, Seongmin Kim, Sanghoon Lee, Jae Yun Song

**Affiliations:** 1 Department of Medical Science, Korea University Graduate School, Seoul 02841, Republic of Korea; 2 Department of Obstetrics and Gynecology, Korea University College of Medicine, Seoul 02841, Republic of Korea

**Keywords:** cell-free DNA, liquid biopsy, ovarian cancer, tumor heterogeneity, UMI sequencing, molecular residual disease

## Abstract

High-grade serous ovarian carcinoma (HGSOC) is characterized by profound genomic instability and spatial heterogeneity. Liquid biopsy, utilizing circulating tumor DNA (ctDNA), offers a non-invasive approach to capture the comprehensive mutational landscape of the disease. This pilot study evaluated the concordance of genomic alterations between cell-free DNA (cfDNA) and matched tumor tissue in patients with HGSOC. Twelve patients with HGSOC undergoing primary cytoreductive surgery were enrolled. Using the Macrogen^®^ Axen™ Cancer Panel 2 with unique molecular identifier (UMI) technology for error suppression, we achieved a theoretical limit of detection of ~0.36% VAF. The mean cfDNA concentration was 107.3 ng/mL, showing a significant positive correlation with FIGO stage (*p* = 0.016). While the sensitivity of cfDNA to detect tissue-confirmed mutations was 57.6%, the overall gene-level concordance was 95.3%, largely driven by negative agreement in wild-type genes. Liquid biopsy revealed a significantly broader mutational spectrum (mean 9.67 alterations/patient) compared to tissue (5.50/patient). Crucially, concordant mutations exhibited high variant allele frequencies (VAFs) (mean 41.4%), whereas plasma-unique discordant mutations showed significantly lower VAFs (mean 7.31%, *p* < 0.001). These preliminary findings suggest that while tissue biopsy likely reflects the dominant clonal population, liquid biopsy may serve as a potential molecular mirror, capturing subclonal variants from spatially distinct metastatic sites and hypoxic niches.

## 1. Introduction

Ovarian cancer is a genetically complex malignancy characterized by substantial intra- and inter-tumoral heterogeneity [1]. This heterogeneity poses a challenge for traditional tissue biopsies, which sample only a portion of a tumor lesion and may miss subclonal mutations present elsewhere. Circulating tumor DNA (ctDNA), the tumor-derived fraction of cell-free DNA (cfDNA) shed into the bloodstream, has emerged as a powerful tool to non-invasively capture a more comprehensive mutational landscape of cancer [2]. Unlike a single-site biopsy, ctDNA reflects DNA contributions from multiple tumor regions and metastatic sites, effectively capturing the spatial heterogeneity of malignant disease [3]. Studies have shown that ctDNA often contains a broader spectrum of mutations than matched tumor tissue, presumably because it aggregates genetic alterations from diverse tumor subclones. For example, comparative analyses have demonstrated that ctDNA can detect additional mutations not found in the primary tumor biopsy due to sampling bias, thereby providing a more complete genomic profile of the cancer [4]. This comprehensive profiling is especially valuable in ovarian carcinoma, where high-grade serous tumors frequently harbor multiple subclonal populations and evolve over time under treatment pressure [5]. In the context of precision oncology, ctDNA offers several advantages. First, it enables longitudinal monitoring of tumor genetics through sequential blood draws, allowing clinicians to track clonal evolution and emerging resistance mutations in real time [6]. Second, by capturing tumor heterogeneity, ctDNA can reveal low-frequency driver mutations or actionable targets that might be missed by analyzing a single tissue sample [7]. This is particularly relevant for selecting targeted therapies or combination treatments tailored to the full complement of a patient’s tumor mutations. Moreover, ctDNA analysis is minimally invasive and can be repeated, which is crucial for ovarian cancer patients who often undergo multiple lines of therapy and may not be amenable to repeated surgical biopsies. Together, these features position ctDNA as a complementary approach to tissue biopsy for comprehensive genomic profiling and personalized treatment planning [8]. An important consideration in ctDNA-based profiling is the ability to distinguish somatic mutations from inherited germline mutations. Ovarian cancer provides a clear example of why this distinction is critical: approximately 13–15% of ovarian carcinomas carry germline BRCA1/2 mutations, while another ~5–7% have somatic BRCA1/2 mutations acquired in the tumor [9]. Identifying a BRCA mutation in a cfDNA sample could imply either an inherited predisposition or a tumor-confined alteration, with very different clinical implications [10]. Germline mutations confer hereditary cancer risk and warrant genetic counseling, whereas somatic mutations are relevant only to the tumor’s biology and therapy response [11]. ctDNA analysis inherently enriches for tumor-derived DNA, but because blood plasma contains a mix of tumor and normal cfDNA, sequencing results must be interpreted carefully to avoid misattributing germline variants as somatic. In practice, this can be addressed by parallel sequencing of a matched normal sample (e.g., leukocyte DNA) or by examining the variant allele fraction (VAF) in plasma. Heterozygous germline variants tend to appear at ~50% allele fraction in cfDNA, reflecting their presence in constitutional DNA, whereas somatic ctDNA mutations usually manifest at lower allele fractions due to dilution by wild-type DNA [10]. Using such approaches, ctDNA studies can effectively differentiate inherited mutations from those arising in the tumor. This ensures that only true tumor-specific mutations inform therapeutic decisions, while any incidental germline findings can be confirmed and acted upon separately. Overall, the integration of ctDNA profiling in ovarian cancer promises to augment our understanding of tumor heterogeneity and improve the accuracy of genomic interpretation by clarifying the somatic versus germline origin of mutations [12].

Few studies have rigorously evaluated the concordance of gene alterations between cfDNA and tissue samples using simultaneous NGS analysis [13]. Common genomic alterations include copy number variations, such as amplification or deletion of genomic regions. the genomic sequencing using NGS technology provided opportunity to characterize genome-wide copy number variations with great resolution. These genomic events observed include mutations in the protein-coding genes, which lead to either activation of oncogenes or inactivation of tumor suppressors. This pilot study investigates the genomic concordance between cfDNA and matched tissue in HGSOC using a hybridization capture-based NGS panel with unique molecular identifier (UMI) error correction. We test the hypothesis that liquid biopsy serves as a “Molecular Mirror,” faithfully reflecting truncal driver mutations while simultaneously revealing subclonal diversity missed by tissue sampling.

## 2. Results

### 2.1. Patient Characteristics and Amount of cfDNA

The study cohort comprised 12 patients with HGSOC (Stage IC3 to IVa). The mean cfDNA concentration was 107.3 ng/mL. A significant positive correlation was observed between cfDNA levels and FIGO stage advancement (Spearman’s ρ = 0.68, *p* = 0.016), supporting the role of cfDNA as a biomarker of total tumor burden. The presence of malignant cell on peritoneal fluid cytology showed no significant correlation with the amount of cfDNA (*p* = 0.905).

### 2.2. Genomic Landscape and Concordance

NGS analysis identified a total of 116 genomic alterations in cfDNA (mean 9.67/patient) and 66 alterations in matched tissue (mean 5.50/patient) (Table 1). The complete list of all detected genomic alterations, including their genomic coordinates and variant allele frequencies (VAFs), is provided in Supplementary Table S1. There were 38 concordant mutations identified in both platforms. The overall concordance rate (including negative agreement) was 95.3%. While this figure appears impressively high, it is important to interpret it with caution. This metric is largely driven by “negative agreement”—the vast number of genes in the 196-gene panel that were wild-type (unmutated) in both tissue and plasma. The sensitivity of cfDNA to detect tissue-confirmed mutations was 57.6%. Conversely, only 32.76% of the mutations found in cfDNA were also found in the tissue. This implies that over 67% of the mutations detected in the liquid biopsy were unique to the plasma and not present in the single-site tissue biopsy.

**Figure 1:**
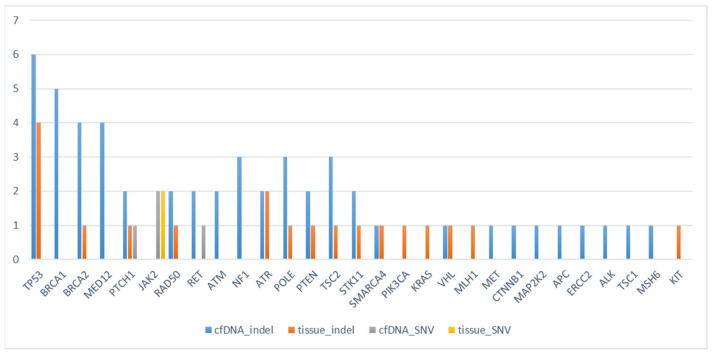
Distribution of Pathogenic Genomic Alterations. The bar chart illustrates the frequency of pathogenic and likely pathogenic variants identified in the study cohort, categorized by sample source and mutation type. TP53 was the most frequently altered gene.

**Figure 2:**
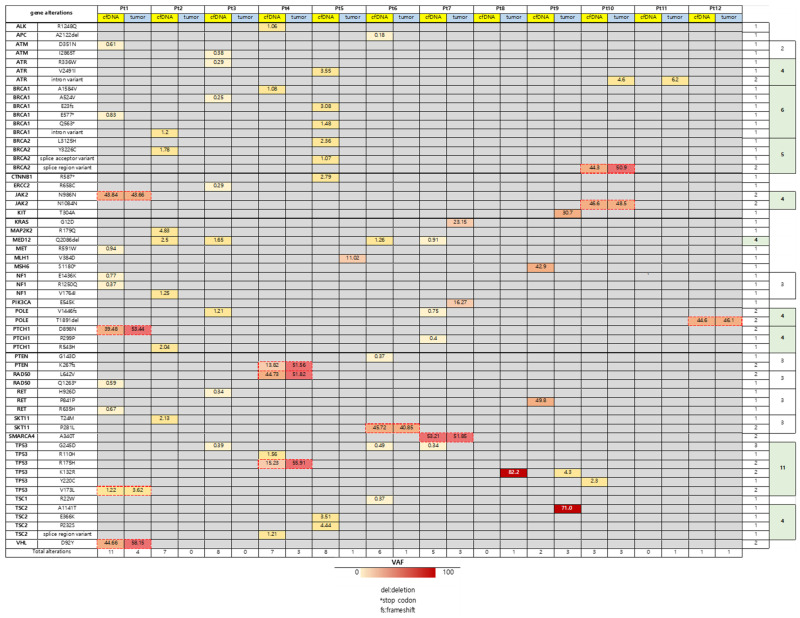
Oncoprint Visualization of Mutational Landscape. A comprehensive oncoprint chart detailing the specific genomic alterations detected across the 12 HGSOC patients using the 196-gene panel. Rows correspond to individual genes, and columns correspond to individual patients.

However, cfDNA detected significantly more unique mutations than tissue, suggesting detection of spatial heterogeneity. There were 29 types of genes altered in study population. Most common altered gene was TP53, followed by BRCA1, and BRCA2 (Figure 1). After disregarding mutations considered as ‘benign’ or ‘likely benign’, 57 and 21 mutations were selected for analysis from cfDNA and tumor, respectively. There were 12 concordant mutations between two platforms from this subset Among 22 pathogenic gene alterations, 5 (22.73%) genes were concordant. On the other hand, there were 7 (15.91%) concordant mutations of variants of unknown significance. Type of mutation was mostly indels, but there were also some synonymous number variants. Figure 2 is an oncoprint chart which shows the gene alterations of each patient.

### 2.3. Variant Allele Frequency (VAF) Analysis

**Figure 3:**
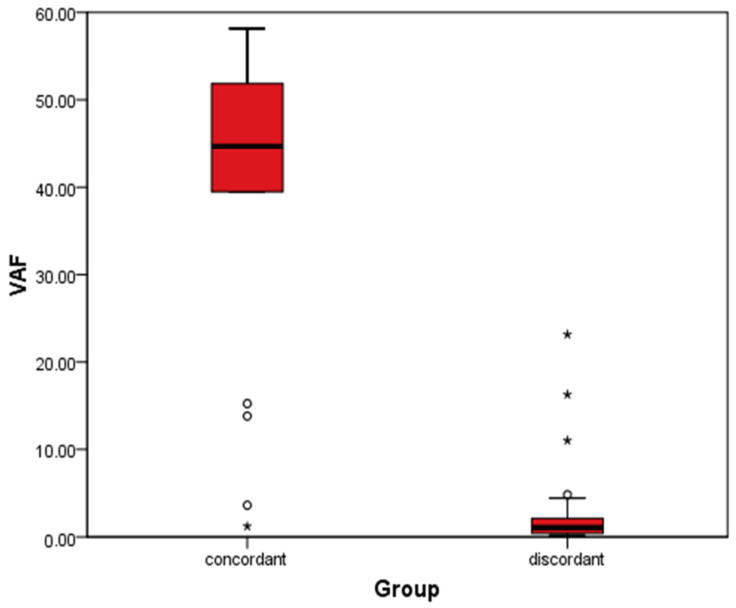
Comparative Analysis of Variant Allele Frequency (VAF). A box-and-whisker plot comparing the distributions of VAF for concordant mutations (detected in both tissue and plasma) versus discordant mutations (detected in only one source). The “Concordant” group exhibits a high median VAF (~41%), indicating these are likely truncal, clonal driver events present throughout the tumor burden. The “Discordant” group exhibits a significantly lower median VAF (~7%), suggesting these variants arise from subclonal populations or spatially distinct metastatic sites with lower DNA shedding contributions. The statistically significant difference (*p* < 0.001) suggests discordant mutations in liquid biopsy may reflect true biological heterogeneity, although technical factors or Clonal Hematopoiesis of Indeterminate Potential (CHIP) cannot be completely excluded.

The mean VAF of concordant mutations was 41.4%, significantly higher than the mean VAF of discordant mutations (7.31%) (*p* < 0.001). This indicates that high-VAF mutations represent clonal (truncal) events shared across the tumor burden, while low-VAF discordant mutations likely represent subclonal populations or spatially distinct metastases detected only in plasma or specific tissue regions (Figure 3). When evaluating the diagnostic accuracy of each cfDNA mutation in relation to tissue mutation, the sensitivity ranged from 58% to 100%, and specificity varied between 50% and 100% (Table 2). The NPV (negative predictive value) was generally high, ranging from 80% to 100%.

## 3. Discussion

This pilot study suggests the potential clinical utility of liquid biopsy in HGSOC. We observed that cfDNA analysis via the Axen Cancer Panel 2 not only detects core truncal mutations (high concordance in *TP53*) but also reveals a broader mutational repertoire than single-site tissue biopsy. The positive correlation observed between cfDNA concentration (mean 107.3 ng/mL) and disease stage suggests that cfDNA levels may reflect the overall tumor burden. This can be attributed to increased tumor cell turnover, apoptosis, or necrosis in advanced stages, leading to a greater release of DNA into the bloodstream, a finding consistent with previous reports. Although we did not find a significant correlation between the amount of cfDNA and the presence of malignant cells in peritoneal fluid cytology (*p* = 0.905), this may be due to the limited sample size. The clear association with stage supports the potential utility of quantitative cfDNA analysis as a non-invasive tool for prognostication and monitoring.

The results of the genomic alteration analysis require nuanced interpretation. While the overall concordance rate across the 196 genes analyzed was high at 95.3%, this figure is largely driven by negative agreement—the absence of mutations in specific genes in both platforms. A more clinically relevant metric, the sensitivity of cfDNA to detect tissue-confirmed mutations, was moderate at 57.58%. This indicates that a substantial proportion of mutations identified in the tissue were not detected in the corresponding cfDNA. This can occur when tumors shed low amounts of DNA (low shedding) or when the VAF of the specific mutation falls below the analytical limit of detection of the assay [14]. Regarding TP53, the observed sensitivity of 50% (Table 2) suggests that even for truncal drivers, detection in plasma can be limited by low tumor fraction in specific patients.

Conversely, only 32.76% of the alterations detected in cfDNA were also identified in the matched tumor tissue. Notably, a substantially higher number of mutations were detected in cfDNA compared to the tissue samples. This bidirectional discordance should not be interpreted as an assay failure but rather as a reflection of the biological phenomenon of tumor heterogeneity. HGSOC is characterized by marked spatial heterogeneity [15]. Single-site tissue biopsies are subject to sampling bias, capturing genetic information only from the specific region sampled. In contrast, cfDNA represents an aggregate of DNA fragments released from the primary tumor and multiple metastatic sites, thereby providing a more comprehensive representation of the cancer’s overall genomic diversity [16]. Therefore, mutations detected exclusively in cfDNA likely originate from subclones present in lesions not sampled during the biopsy, demonstrating that liquid biopsy is a potent complementary tool for assessing tumor heterogeneity beyond the limitations of tissue biopsy.

The observation that cfDNA analysis yielded nearly double the number of mutations per patient compared to tissue (9.67 vs. 5.50) is the most clinically significant finding of this report. This “bidirectional discordance” reflects the fundamental biology of HGSOC. Recent studies have shown that chemotherapy pressure can induce clonal evolution, leading to the emergence of resistant subclones that may be spatially distinct [17]. The 42% of mutations found in tissue but missed in plasma can be attributed to “low shedding” phenotypes. Not all tumors release DNA with equal efficiency [14]. The “blood-peritoneum barrier” is a specific challenge in ovarian cancer; large metastatic burdens confined to the peritoneal cavity may shed DNA into ascites fluid that does not fully equilibrate with the systemic circulation [18]. Furthermore, if a mutation is subclonal within the biopsied tissue itself, its representation in the total plasma pool may fall below the limit of detection (LOD) of even deep sequencing assays [19]. The mutations unique to plasma strongly support the concept of spatial heterogeneity. HGSOC is a spatially heterogeneous disease. A biopsy of the ovary does not reflect the genomic profile of a metastasis in the liver or omentum. The plasma, receiving drainage from all these sites, aggregates the mutational signals. Thus, liquid biopsy effectively “samples” the entire body. Identification of these subclonal mutations is critical because they often harbor the seeds of therapeutic resistance [20].

The VAF analysis provides a robust framework for distinguishing truncal from subclonal mutations [21]. The high VAF of concordant TP53 mutations is consistent with the characteristics of early, truncal driver events, although germline variants or Clonal Hematopoiesis of Indeterminate Potential (CHIP) cannot be fully ruled out without matched normal controls. In contrast, the low-VAF variants found only in plasma likely represent “private” mutations from distant metastases. This distinction has profound implications for monitoring resistance. For example, in patients treated with PARP inhibitors, resistance often develops via “reversion mutations” in *BRCA1* or *BRCA2* genes. These secondary mutations restore the reading frame of the gene, restoring functional homologous recombination and rendering the tumor resistant to the drug. These reversion events typically start as small subclonal populations. Liquid biopsy has been shown to detect these low-VAF reversion mutations months before clinical recurrence becomes evident, offering a window for early therapeutic intervention [22]. Tissue biopsy, unless repeated on the specific progressing lesion would miss these emerging clones [20].

A major technical challenge in liquid biopsy is the “biological background noise.” Not all mutations in cfDNA are tumor-derived. Inherited mutations (e.g., germline *BRCA1/2*) will appear in cfDNA at a VAF of ~50%. Distinguishing these from somatic tumor mutations requires matched normal control sequencing (e.g., white blood cells) or careful bioinformatic inference [23]. As individuals age, hematopoietic stem cells acquire somatic mutations (commonly in *TP53*, *DNMT3A*, *ASXL1*) that confer a survival advantage, leading to a clonal expansion of blood cells. These mutations release “normal” DNA into the plasma that carries these variants. Without matched WBC sequencing, a CHIP mutation in *TP53* could easily be misidentified as a tumor mutation, leading to a false-positive diagnosis or incorrect monitoring results [10]. The pilot study’s limitation of not sequencing matched WBCs highlights the absolute necessity of this control step in future clinical protocols.

The strong correlation between cfDNA levels and FIGO stage supports the use of quantitative cfDNA as a biomarker for tumor burden [24]. Beyond quantification, qualitative analysis (mutation detection) holds promise for early detection. Previous studies have demonstrated that multi-analyte liquid biopsies (combining mutations and proteins) can detect early-stage ovarian cancers with reasonable sensitivity, although sensitivity in Stage I remains a hurdle [25]. For monitoring, the dynamic range of liquid biopsy allows for the assessment of “molecular response.” A rapid drop in ctDNA levels after surgery or chemotherapy indicates effective debulking and treatment response. Conversely, the persistence of ctDNA (Molecular Residual Disease, MRD) is a powerful predictor of inevitable relapse, often preceding CA-125 elevation or radiological progression by months [20].

This study possesses several important strengths. First, patients were enrolled prospectively, and the inclusion was restricted to a histologically homogeneous cohort of HGSOC patients, enhancing the clarity of result interpretation. Second, the acquisition of matched blood and tissue samples at the pre-treatment stage ensured temporal consistency between the samples and eliminated confounding effects induced by therapy. Third, the use of a standardized NGS platform (Macrogen^®^ Axen™ Cancer Panel 2) for analyzing all samples minimized technical variability. Finally, the study empirically demonstrated a significant correlation between quantitative cfDNA data and clinical stage, highlighting the potential of cfDNA as a biomarker reflecting tumor burden.

Despite these strengths, this study has several important limitations. First, as a pilot study with a small cohort (N = 12), the statistical power is limited, and caution must be exercised when generalizing the findings. Second, the absence of matched normal (white blood cell) sequencing is a critical limitation. Although we applied rigorous bioinformatics filters (e.g., gnomAD) to exclude common germline polymorphisms, we cannot definitively distinguish whether variants detected in cfDNA—especially those with high VAFs or in genes like TP53—are true tumor-derived somatic mutations, rare germline variants, or attributable to CHIP. Consequently, the high concordance VAFs observed may partially reflect germline contributions. Future studies must incorporate matched normal controls to strictly define somatic origin. Third, samples were collected only at a single time point before surgery, precluding the assessment of temporal heterogeneity, clonal evolution under treatment pressure, or minimal residual disease. Fourth, the use of a targeted panel comprising 196 genes meant that other genomic alterations or structural variations outside the panel scope could not be evaluated.

## 4. Materials and Methods

### 4.1. Study Population

The biospecimens analyzed in this study were retrospectively obtained from the Korea University Anam Hospital Human Biobank. The samples were originally collected from patients with HGSOC who underwent primary cytoreductive surgery. All donors provided written informed consent for the donation and use of their biological resources for research purposes at the time of collection. The current study protocol involving the use of these anonymized samples was approved by the Institutional Review Board of Korea University Anam Hospital (No. 2021AN0019) with a waiver of informed consent, in accordance with Article 16 of the Bioethics and Safety Act [26].

### 4.2. Sample Acquisition and DNA Extraction

For acquisition of plasma ctDNA, peripheral blood (15 mL) was collected in EDTA tubes one day prior to surgery. Plasma was isolated within 12 h of collection using a two-step centrifugation protocol (2000× *g* for 20 min, followed by 3200× *g* for 30 min) to remove cellular debris and stored at −80 °C until extraction. ctDNA was extracted from 2 to 4 mL of plasma using the QIAamp Circulating Nucleic Acid Kit (Qiagen, Hilden, Germany) following the manufacturer’s instructions for the enrichment of low-molecular-weight circulating nucleic acids. DNA concentration was quantified using the Qubit dsDNA HS Assay Kit (Thermo Fisher Scientific, Waltham, MA, USA). To acquire tissue DNA, formalin-fixed, paraffin-embedded (FFPE) tumor tissue blocks were obtained from surgical specimens. Genomic DNA was extracted from tumor-rich sections using the QIAamp DNA FFPE Tissue Kit (Qiagen), following deparaffinization and proteinase K digestion.

### 4.3. Targeted Next-Generation Sequencing

Targeted sequencing was performed using the Macrogen^®^ Axen™ Cancer Panel 2, a hybridization capture-based NGS panel targeting the coding exons and selected introns of 196 cancer-related genes. DNA libraries were constructed using the Agilent SureSelect XT target enrichment system (Agilent Technologies, Santa Clara, CA, USA). Genomic DNA was fragmented (for tissue samples), end-repaired, adenylated, and ligated with indexed adapters. The libraries were then hybridized with biotinylated RNA probes specific to the 196 target genes and captured using streptavidin beads. Prepared libraries were pooled and sequenced on an Illumina NextSeq 500 platform (Illumina, San Diego, CA, USA) to achieve high depth of coverage (targeting >1000× for tissue and >2000× for cfDNA) to ensure high sensitivity for low-frequency somatic variants.

### 4.4. Bioinformatics and Statistical Analysis

Raw sequencing data were processed using the Macrogen Axen™ System pipeline. Unique Molecular Identifiers (UMIs) were utilized to group PCR duplicates into consensus families, effectively suppressing sequencing errors and PCR bias. Variants were called using Mutect2 with a Panel of Normals (PoN) to filter technical artifacts.

Quality Control (QC) criteria were as follows: (1) Mean effective sequencing depth of >1000× for tissue and >2000× for cfDNA; (2) On-target rate > 90%; (3) Coverage uniformity > 80%; and (4) Phred quality score (Q30) > 80%.

To ensure clarity, concordance was analyzed at two levels: (1) Gene-level Concordance—defined as the agreement on the presence or absence of any mutation within a specific gene between paired samples (including negative agreement)—and (2) Variant-level Concordance—defined as the detection of the identical genomic alteration (same chromosome, coordinate, reference, and alternate allele) in both cfDNA and tissue. To filter common germline polymorphisms in the absence of matched WBCs, we excluded variants with >1% population allele frequency in gnomAD and 1000 Genomes databases. It is noted that this population-based filtering does not remove rare germline variants or CHIP. Variants were annotated using ClinVar and ACMG guidelines.

Comparisons of VAF between groups were performed using Student’s *t*-test. It should be noted that variants are nested within patients; however, given the exploratory nature of this pilot study, variants were treated as independent observations for these comparisons.

## 5. Conclusions

This comprehensive analysis establishes liquid biopsy as a potent, complementary tool in the management of High-Grade Serous Ovarian Carcinoma. While tissue biopsy remains necessary for initial histological diagnosis, it is insufficient for capturing the full spatial and temporal complexity of the disease. The concordance data reveals that liquid biopsy detects high-VAF mutations consistent with truncal driver mutations (like *TP53*) while simultaneously uncovering a vast reservoir of subclonal, discordant mutations that reflect the true systemic burden of the cancer.

By acting as a “molecular mirror,” circulating tumor DNA allows clinicians to peer into the evolutionary dynamics of the tumor in real-time, offering opportunities to detect resistance mechanisms like *BRCA* reversions before they manifest clinically. Moving forward, the routine integration of matched white blood cell sequencing to filter CHIP, coupled with multi-omic approaches (methylation and fragmentomics), will be essential to unlock the full potential of this technology. Liquid biopsy is not merely a convenient alternative to surgery; it is a biological imperative for the era of precision oncology.

## Figures and Tables

**Table 1:** Patient-level comparison of mutations in cfDNA and tumor tissue. The table details the number of mutations identified in each sample type, the count of concordant alterations, the overall concordance rate, and the sensitivity of cfDNA for detecting tissue-confirmed mutations for each patient.

Patient ID	cfDNA Mutations	Tissue Mutations	Concordant Mutations	Concordance Rate (%) ^1^	Tissue Alterations in cfDNA (%) ^2^	cfDNA Alterations in Tissue (%) ^3^	Peritoneal Washing Cytology	Amount of cfDNA
Pt1	16	7	6	94.36%	85.71%	37.50%	Positive	225.60
Pt2	21	8	2	87.18%	25.00%	9.52%	Positive	250.15
Pt3	16	5	5	91.79%	100.00%	31.25%	Negative	91.50
Pt4	17	5	5	91.28%	50.00%	29.41%	Negative	109.40
Pt5	15	5	4	93.85%	80.00%	26.67%	Positive	42.95
Pt6	8	7	3	95.38%	42.86%	37.50%	Positive	78.50
Pt7	12	9	7	96.41%	77.78%	58.33%	Positive	200.40
Pt8	1	2	1	99.49%	50.00%	100.00%	Positive	27.64
Pt9	3	6	0	95.38%	0.00%	0.00%	Negative	110.50
Pt10	4	3	2	98.46%	66.67%	50.00%	Negative	24.52
Pt11	2	1	1	99.49%	66.67%	100.00%	Negative	38.44
Pt12	1	1	1	100.00%	100.00%	100.00%	Positive	88.00
Total	116	66	38	95.30%	57.58%	32.76%	N/A	1287.60
Mean	9.67	5.5						107.30

^1^ Concordance Rate: The percentage of genes (out of 196) where the presence or absence of a mutation was identical between the cfDNA and tissue samples. ^2^ % of tissue alterations found in cfDNA (Sensitivity): The proportion of mutations detected in tissue that were also identified in cfDNA. ^3^ % of cfDNA alterations found in tissue: The proportion of mutations detected in cfDNA that were also identified in tissue.

**Table 2:** Diagnostic performance of cell-free DNA (cfDNA) mutations.

cfDNA Mutations		Tissue Mutations		Sensitivity (%)	Specificity (%)	PPV (%)	NPV (%)	Diagnostic Accuracy (%)
		+	−					
TP53	+	2	4	50	50	33.33	66.67	50
	−	2	4					
BRCA1	+	0	5	N/A	58.33	N/A	100	58.33
	−	0	7					
BRCA2	+	1	2	100	81.82	33.33	100	83.33
	−	0	9					
MED12	+	0	4	N/A	66.67	N/A	100	66.67
	−	0	8					
ATR	+	0	2	0	80	0	80	66.67
	−	2	8					
JAK2	+	2	0	100	100	100	100	100
	−	0	10					
POLE	+	1	2	100	81.82	33.33	100	83.33
	−	0	9					
PTCH1	+	1	2	100	81.82	33.33	100	83.33
	−	0	9					
TSC2	+	0	2	0	81.82	0	90	75
	−	1	9					
total	+	7	23	58.33	76.04	23.33	93.59	74.07
	−	5	73					

Note: N/A indicates that Sensitivity or PPV could not be calculated because no somatic mutations were detected in the reference sample (tissue or cfDNA) for this specific gene in the cohort.

## Data Availability

The raw data supporting the conclusions of this article will be made available by the authors on request.

## Supplementary Materials

The following supporting information can be downloaded at: https://www.mdpi.com/article/10.3390/ijms27031305/s1.
