# Supplementary material for: Concordance of Genomic Alterations in Ovarian Cancer Tissues and Circulating-Tumor DNA: A Pilot Study
Source: Int J Mol Sci. 2026 Jan 28;27(3):1305. doi: 10.3390/ijms27031305 (PMC12897642; doi:10.3390/ijms27031305)
Supplement: Supplementary file 1 [file ijms-27-01305-s001.zip › ijms-4056495-supplementary.pdf]

International Journal of
Molecular Sciences

MDPI

Supplementary Materials

Supplementary Table S1. List of putative somatic genomic alterations detected in cell-free DNA and matched tumor tissue. This table details the specific genomic characteristics of all somatic mutations identified in the study cohort (N = 12). Variants are classified into two categories: Concordant (identical variants detected in both cfDNA and tissue) and discordant (variants detected exclusively in either cfDNA or tissue). Genomic coordinates are based on the hg19 reference genome.

Patient	Gene	Genomic Coordinate	cDNA Change	Protein Change	Tissue VAF(%)	cfDNA VAF (%)	Classification
**Pt1**	TP53	chr17:7578413	c.517G>T	p.V173L	3.62	1.22	Concordant
	VHL	chr3:10183805	c.274G>T	p.D92Y	58.15	44.66	
	JAK2	chr9:5090810	c.2958C>T	p.N986N	43.66	43.84	
	PTCH1	chr9:9822414	c.2692G>A	p.D898N	53.44	39.48	
**Pt4**	PTEN	chr10:89717769	c.800delA	p.K267fs	51.56	13.82	
	TP53	chr17:7578406	c.524G>A	p.R175H	55.91	15.23	
	RAD50	chr5:131930691	c.1924T>G	p.L642V	51.82	44.73	
**Pt6**	STK11	chr19:1221319	c.842C>T	p.P281L	40.85	45.72	
**Pt7**	SMARCA4	chr19:11098500	c.1018G>A	p.A340T	51.85	53.21	
**Pt10**	BRCA2	chr13:32914288	c.475+1G>T	p.?	50.9	44.3	
	JAK2	chr9:5090810	c.3252T>C	p.Asn1084Asn	48.5	46.6	
**Pt12**	POLE	chr12:133229385	c.5672_5674delCCA	p.Thr1891del	46.1	44.6	
**Pt1**	RAD50	chr5:131977904	c.3787C>T	p.Q1263*	0	0.59	Discordant
	BRCA1	chr17:41245819	c.1729G>T	p.E577*	0	0.83	
	MET	chr7:116395478	c.1771C>T	p.R591W	0	0.94	
	RET	chr10:43609952	c.1904G>A	p.R635H	0	0.67	
	ATM	chr11:108117840	c.1051G>A	p.D351N	0	0.61	
	NF1	chr17:29562669	c.3749G>A	p.R1250Q	0	0.37	
	NF1	chr17:29585494	c.4306G>A	p.E1436K	0	0.77	
**Pt2**	PTCH1	chr9:98238416	c.1628G>A	p.R543H	0	2.04	
	BRCA2	chr13:32972327	c.9677A>C	p.Y3226C	0	1.78	
	NF1	chr17:29654538	c.5290G>A	p.V1764I	0	1.25	
	BRCA1	chr17:41228495	c.4484+10A>G	p.?	0	1.2	
	STK11	chr19:1206983	c.71C>T	p.T24M	0	2.13	
	MAP2K2	chr19:4101271	c.536G>A	p.R179Q	0	4.83	
	MED12	chrX:70360679	c.6256_6258delCAG	p.Q2086del	0	2.5	
**Pt3**	TP53	chr17:7577547	c.734G>A	p.G245D	0	0.39	
	ERCC2	chr19:45855838	c.1972C>T	p.R658C	0	0.29	
	ATR	chr3:142281238	c.1006C>T	p.R336W	0	0.29	
	RET	chr10:43617439	c.2776C>G	p.H926D	0	0.34	
	ATM	chr11:108218015	c.8594T>C	p.I2865T	0	0.38	
	POLE	chr12:133220098	c.4337_4338delTG	p.V1446fs	0	1.21	
	BRCA1	chr17:41245977	c.1571C>T	p.A524V	0	0.25	
	MED12	chrX:70360679	c.6256_6258delCAG	p.Q2086del	0	1.65	
**Pt4**	ALK	chr2:29436850	c.3743G>A	p.R1248Q	0	1.06	
	TSC2	chr16:2136191	c.4663-3C>T	p.?	0	1.21	
	TP53	chr17:7579358	c.329G>A	p.R110H	0	1.56	
	BRCA1	chr17:41223180	c.4751C>T	p.A1584V	0	1.08	
**Pt5**	CTNNB1	chr3:41277290	c.1759C>T	p.R587*	0	2.79	
	BRCA2	chr13:32900377	c.476-2A>G	p.?	0	1.07	
	BRCA1	chr17:41245861	c.1687C>T	p.Q563*	0	1.48	
	BRCA1	chr17:41276044	c.68_69delAG	p.E23fs	0	3.08	

	ATR	chr3:142177832	c.7471G>A	p.V2491I	0	3.55
	BRCA2	chr13:32968943	c.9374T>A	p.L3125H	0	2.36
	TSC2	chr16:2106690	c.694C>T	p.P232S	0	4.44
	TSC2	chr16:2110791	c.1096G>A	p.E366K	0	3.51
	MLH1	chr3:37067240	c.1151T>A	p.V384D	11.02	0
**Pt6**	TSC1	chr9:135804196	c.64C>T	p.R22W	0	0.37
	TP53	chr17:7577547	c.734G>A	p.G245D	0	0.49
	APC	chr5:112177642	c.6363_6365delTGC	p.A2122del	0	0.18
	PTEN	chr10:89692944	c.428G>A	p.G143D	0	0.37
	MED12	chrX:70360679	c.6256_6258delCAG	p.Q2086del	0	1.26
**Pt7**	TP53	chr17:7577547	c.734G>A	p.G245D	0	0.34
	PTCH1	chr9:98242720	c.897G>A	p.P299P	0	0.4
	POLE	chr12:133220098	c.4337_4338delTG	p.V1446fs	0	0.75
	MED12	chrX:70360679	c.6256_6258delCAG	p.Q2086del	0	0.91
	PIK3CA	chr3:178936091	c.1633G>A	p.E545K	16.27	0
	KRAS	chr12:25398284	c.35G>A	p.G12D	23.15	0
**Pt8**	TP53	chr17:7578413	c.395A>G	p.Lys132Arg	82.2	0
**Pt9**	MSH6	chr2:4802821	c.3539C>G	p.Ser1180*	0	42.9
	RET	chr10:43615175	c.2523G>A	p.Pro841Pro	0	49.8
	KIT	chr4:55593655	c.910A>G	p.Thr304Ala	30.7	0
	TSC2	chr16:2112341	c.3421G>A	p.Ala1141Thr	71	0
	TP53	chr17:7578413	c.395A>G	p.Lys132Arg	4.3	0
**Pt10**	TP53	chr17:757819	c.659A>G	p.Tyr220Cys	0	2.3
	ATR	chr3:142261023	c.4153-10dupT	p.?	4.6	0
**Pt11**	ATR	chr3:142261023	c.4153-10dupT	p.?	6.2	0

Abbreviations: VAF, Variant Allele Frequency; cfDNA, cell-free DNA; SNV, Single-Nucleotide Variant; Indel, Insertion/Deletion. Note: To ensure strict patient de-identification and compliance with biobank privacy protocols for retrospective samples, data are presented as a pooled cohort list rather than individual patient profiles. A VAF of 0 indicates that the variant was not detected above the reporting threshold in that compartment.

https://doi.org/10.3390/ijms27031305
